# Docetaxel induces apoptosis in hormone refractory prostate carcinomas during multiple treatment cycles

**DOI:** 10.1038/sj.bjc.6603129

**Published:** 2006-05-09

**Authors:** G Kramer, S Schwarz, M Hägg, A Mandic Havelka, S Linder

**Affiliations:** 1 Department of Urology, University of Vienna, Vienna, Austria; 2 Cancer Center Karolinska, Department of Oncology and Pathology, Karolinska Institute and Hospital, S-171 76 Stockholm, Sweden

**Keywords:** prostate cancer, treatment, apoptosis, biomarker, M30-ELISA

## Abstract

Caspase-cleaved proteins are released from disintegrated apoptotic cells and can be detected in the circulation. We here addressed whether caspase-cleaved cytokeratin 18 (CK18-Asp396) can be used as a serum biomarker for assessment of the clinical efficiency of chemotherapy in hormone-refractory prostate cancer (HRPC). A total of 82 patients with HRPC were evaluated during 751 treatment cycles, either with estramustine (EMP)/vinorelbine or with EMP/docetaxel. The levels of CK18-Asp396 and of total CK18 were measured in patient serum before and during therapy by ELISA. Docetaxel induced significant increases in serum CK18-Asp396 (*P*<0.0001) and total CK18 (*P*<0.0002), suggesting induction of apoptosis. Similarly, vinorelbine induced increases in both CK18-Asp396 and CK18 (*P*<0.001 and 0.011). In contrast, EMP induced increases in total serum CK18 (*P*<0.0001), but not in CK18-Asp396 (*P*=0.13). The amplitudes of docetaxel-induced increases were associated with baseline prostate-specific antigen (PSA) and CK18 serum levels in these patients, consistent with tumoral origin of caspase-cleaved fragments. Docetaxel induced significant increases in CK18-Asp396 during second-, third- and fourth-line therapy and induced increased levels of CK18-Asp396 during treatment cycles 1–8. In contrast, vinorelbine induced significant increases only during cycles 1–3. In a subgroup of 32 patients that received EMP/vinorelbine in second line followed by EMP/docetaxel in third line, docetaxel induced stronger increases than vinorelbine (*P*=0.008). These results show that the CK18-Asp396 serum marker can be used to assess tumour apoptosis *in vivo* and suggest that the clinical efficiency of docetaxel in HRPC is due to induction of apoptosis during multiple treatment cycles.

Although treatment of hormone-refractory prostate cancer (HRPC) is still considered palliative, recent studies have demonstrated improved overall survival using docetaxel, in combination with estramustine phosphate (EMP) or prednisone (Petrylak *et al*, 2004; Tannock *et al*, 2004). Docetaxel is a semisynthetic taxane, a class of anticancer agents that bind to beta tubulin, thereby stabilising microtubules and inducing cell-cycle arrest and apoptosis (Hennequin *et al*, 1995; Pienta, 2001). Estramustine phosphate is a nornitrogen mustard–estradiol conjugate that was developed as an alkylating agent specific for oestrogen receptor-positive cells (Smith, 1999). It has later been shown that EMP interferes with microtubuli dynamics, whereas the alkylating effect is insignificant (Smith, 1999). Estramustine phosphate is often used in combination with vinorelbine (Carles *et al*, 1998; Smith *et al*, 2000), a semisynthetic vinca alkaloid which also inhibits microtubule assembly (Johnson *et al*, 1996; Oudard *et al*, 2001).

Similar to other types of chemotherapy, prostate cancer treatment is based on combinations of drugs that are administered during repetetive cycles and at various stages of clinical progression. The response to treatment is commonly evaluated using the tumour marker PSA (prostate-specific antigen). Prostate-specific antigen levels have been reported to correlate with tumour volume (Partin *et al*, 1990) and pathological stage (Oesterling *et al*, 1988). The overall benefit of monitoring serum PSA after treatment for prostate cancer is, however, controversial (Thalmann *et al*, 1996; Kramer *et al*, 1997; Vicini *et al*, 2005). In the recent TAX327 study, the rates of PSA response and patients survival were discordant between two docetaxel groups (Tannock *et al*, 2004).

Noninvasive assessment of treatment-induced tumour cell death is desirable both for accurate management of patients and for detailed characterisation of different treatment modalities. It is desirable to assess whether all drugs in different combinations are effective and to determine for how many cycles it is meaningful to treat patients before resistance has developed. Cytokeratin 18 (CK18) is a suitable serum biomarker for non-invasive determination of apoptosis of epithelial cells (Linder *et al*, 2004). Cytokeratin 18 is an abundant intracellular protein that is cleaved by caspases during apoptosis, and preclinical and clinical studies have shown that caspase-cleaved CK18 fragments are released from apoptotic cells into the extracellular compartment (Kramer *et al*, 2004; Linder *et al*, 2004). In contrast to other serum biomarkers used to assess tissue damage during cancer treatment such as cytochrome *c* (Renz *et al*, 2001) and nucleosomal DNA (Holdenrieder *et al*, 2001), caspase-cleaved CK18 is produced only during apoptosis and only by epithelially derived cells. Chemotherapy-sensitive cells of the bone marrow do not express CK18. We here used measurements of caspase-cleavage products of CK18 in patient serum to assess the efficiency of different anticancer drugs during prostate cancer treatment. We demonstrate that docetaxel induces significantly increased levels of caspase-cleaved CK18 in patient serum at both early and late stages of prostate cancer progression.

## PATIENTS AND METHODS

### Patients

A total of 82 patients with HRPC were included. Of these, 71 patients were selected for second-line chemotherapy based on two or more consecutive increases in PSA not <14 days apart and/or objective progression of metastases in bone or computed tomography (CT) scan in a state of surgical or chemical castration (Table 1). Antiandrogen therapy was discontinued >4 weeks before inclusion (>6 weeks for bicalutamide). Continued evidence of disease progression was required after anti-androgen withdrawal. In total, 62 patients received sequential chemotherapy with EMP and vinorelbine, and nine patients with EMP and docetaxel. In 32 patients, who progressed on EMP/vinorelbine, treatment was switched to third-line chemotherapy with EMP/docetaxel. In one patient who progressed on third line EMP/docetaxel treatment was switched to EMP/vinorelbine again. In addition to these 71 patients, 11 patients were referred to the Department of Urology, Vienna, for third line EMP/vinorelbine. Ethical approval and consent was obtained for collection of serum samples.

### Chemotherapeutic regimen

Chemotherapy consisted of sequential application of 300 mg EMP intravenously from day 1 to day 3 and vinorelbine (Navelbine^R^; 30 mg m^−2^) or docetaxel (Taxotere^R^; 60 mg m^−2^) on day 5. This regimen was repeated every 3 weeks.

### Assessment of apoptosis

Serum samples were collected and frozen at −70°C. Samples were collected at day 1, 3, 5 and 7 during therapy. A total of 751 cycles were analysed. Blood was not available for all time points (2602 samples were available, yielding a coverage of 87%). Samples were assayed in duplicate for caspase-cleaved CK18 using the M30-Apoptosense™ ELISA kit (PEVIVA AB, Bromma, Sweden) and for total soluble CK18 using the M65-ELISA kit (PEVIVA) as recommended by the manufacturer.

### Statistics

The data sets obtained were not normally distributed and nonparametric statistics were used throughout this study. The Wilcoxon matched-pairs signed-ranks test was used to analyse whether statistically significant increases in CK18 levels occurred during therapy (pairwise comparisons of pre- and post-treatment values). The levels of CK18 are presented as median values with 25–75% spreads. The Wilcoxon two-sample test was used to calculate whether differences in the median levels of CK18 were statistically significant.

## RESULTS

### Increases in the serum levels of caspase-cleaved CK18 fragments during chemotherapy of prostate cancer patients

A total of 82 patients with HRPC were treated with a combination of EMP and vinorelbine, or with EMP and docetaxel, mainly as second- or third-line therapy following hormone ablation (Table 1). Estramustine phosphate was administered on days 1, 2 and 3 (751 therapy cycles evaluated); vinorelbine or docetaxel was administered on day 5 (462 and 289 cycles evaluated, respectively). Caspase-cleaved CK18 fragments (CK18-Asp396; CK18 molecules cleaved at Asp396) were measured in patient serum using the M30-Apoptosense™ ELISA (Kramer *et al*, 2004). We also measured total serum CK18 using the M65-ELISA (Kramer *et al*, 2004). *In vitro* experiments and experiments using mouse xenografts showed maximal release of CK18 molecules at 48 h after drug treatment (Ueno *et al*, 2003; Kramer *et al*, 2004) (our unpublished data). We therefore evaluated the response to EMP between day 1 (prior to therapy) and day 3, and the response to vinorelbine or docetaxel between days 5 and 7.

Docetaxel induced increases in CK18-Asp396 in 71% of all evaluated treatment cycles, vinorelbine in 65% and EMP in 50%. The Wilcoxon matched-pairs signed-ranks Test was used to examine the possible statistical significance of increases in serum CK18 molecules by different agents. Estramustine phosphate did not produce significant increases in CK18-Asp396 (*P*=0.13), whereas highly significant increases in total CK18 were observed (*P*<0.0001). Highly significant increases in CK18-Asp396 were induced by docetaxel (*P*<0.0001) and by vinorelbine (*P*<0.001). Similarly, significant increases in total CK18 were induced by docetaxel (*P*<0.0002) and by vinorelbine (*P*<0.011).

**Figure 1:**
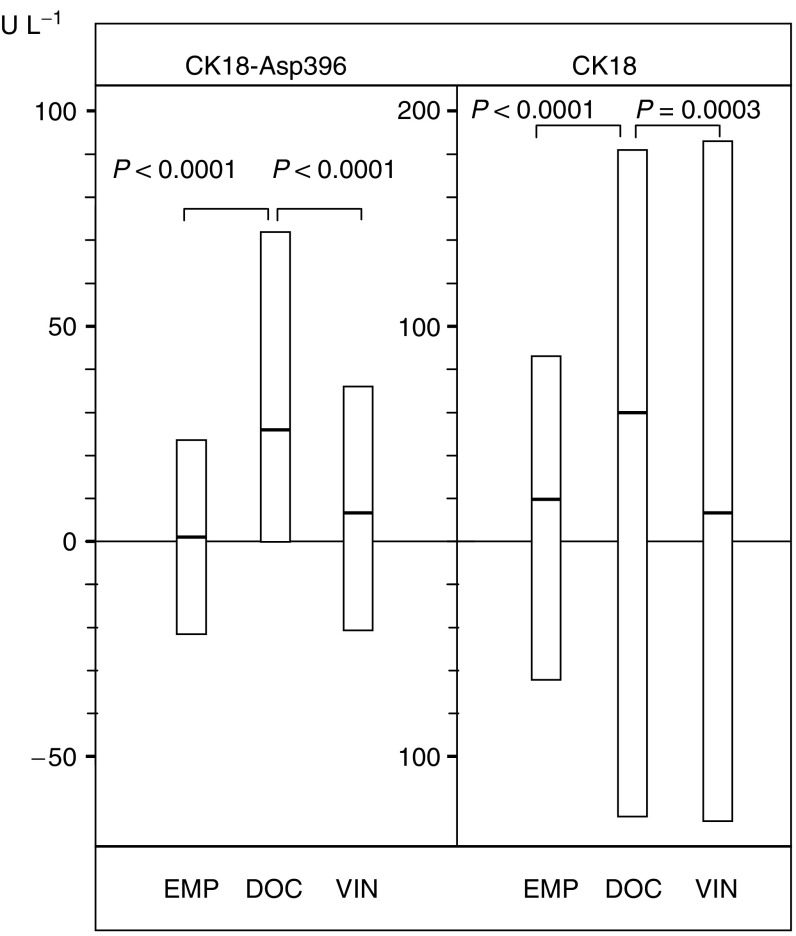
Alterations of serum CK18-Asp396 and CK18 during chemotherapy. Box plots (median with 25th and 75th percentiles) of alterations of pretreatment levels and levels measured after 48 h (days 1–3 for EMP (629 EMP cycles) are shown; days 5–7 for docetaxel (224 cycles) and vinorelbine (343 cycles)). Serum levels of caspase-cleaved CK18 (CK18-Asp396) and CK18 were measured by ELISA.

To obtain quantitative data with regard to the amplitudes of CK18 alterations, median serum CK18 levels were compared before and after onset of treatment. The Wilcoxon two-sample test was used to determine whether differences in the medians were statistically significant. Neither EMP nor vinorelbine induced significant increases in the serum CK18-Asp396 or CK18 by this test (Table 2). In contrast, docetaxel induced a median increase of CK18-Asp396 of 18.7% (*P*=0.015). An increase of a similar amplitude (21.4%) was observed for total CK18 (*P*=0.014) (Table 2). Data were also analysed as increases from pretreatment levels (Figure 1). Only cycles from which both pre- and post-treatment sera were available were included. The result differed only marginally from those presented in Table 2 (based on all data points available). The higher increases of CK18-Asp396 and total CK18 induced by docetaxel compared to other agents were highly significant (Figure 1).

Since patients were treated with a combination of EMP and vinorelbine (or docetaxel) in each treatment cycle, we assessed whether there was an association between the alterations in serum CK18 during these types of treatments. No significant correlation between CK18-Asp396 (or CK18) increases on day 1–3 and on day 5–7 were observed (either for EMP to vinorelbine or EMP to docetaxel), suggesting different mechanisms of cell sensitivity to these agents.

### Increases in CK18-Asp396 during docetaxel and vinorelbine therapy are associated with baseline levels of PSA and CK18

Increases of serum CK18-Asp396 and CK18 levels during therapy could be due to release from dead tumour cells, from normal epithelial cells or from both tumour and normal cells. If CK18 molecules are released from tumour cells during therapy, higher increases (in absolute numbers) are expected in patients with large tumour loads. If, however, CK18 is released as a consequence of toxicity to normal epithelial tissue, increases will not correlate to tumour load. Prostate-specific antigen levels reflect prostate tumour volume, although other factors such as hormonal influences also determine these levels (Crawford *et al*, 1996). Cytokeratin 18 levels, measured with the TPS test, also correlate to prostate cancer tumour load (Tarle *et al*, 1993), and pretreatment CK18-Asp396 levels correlate to tumour load in breast cancer patients (Ueno *et al*, 2003). Significant associations were observed between the amplitudes of docetaxel-induced increases of CK18-Asp396 and baseline levels of PSA, CK18 and CK18-Asp396 (comparing the medians of the highest and lowest quartiles) (Table 3). Significant associations were also observed between docetaxel-induced increases of CK18 and baseline levels of CK18, but not with PSA or CK18-Asp396. These findings suggest an association between docetaxel-induced increases in serum CK18-Asp396 and the presence of tumour. In contrast, vinorelbine- or EMP-induced increases in CK18-Asp396 did not show significant associations to baseline levels of PSA, CK18 and CK18-Asp396 (Table 3).

### Increases in CK18-Asp396 levels during second-line vinorelbine and third-line docetaxel treatment

The data in Table 2 suggest that docetaxel induces stronger increases in serum CK18 than vinorelbine. The present study was, however, not randomised between docetaxel and vinorelbine. We analysed the response to docetaxel and vinorelbine in a subgroup of 32 patients that received both drugs, vinorelbine in second line (169 cycles) followed by docetaxel in third line (158 cycles). The alterations from baseline levels were analysed. Monitoring serum PSA indicated that docetaxel was more efficient than vinorelbine (Table 1). Consistent with the higher efficiacy of docetaxel, a 5.3% increase in serum CK18-Asp396 was observed in second line and a 16.2% increase in third line in these 32 patients (Table 4). The higher increase by docetaxel was statistically significant (*P*=0.008). A similar result was observed for total CK18. The response to EMP was in the range of 3–5% increase of CK18-Asp396 and CK18 in these patients during second- and third-line treatment. Higher baseline levels were observed in DOC-treated patients, probably reflecting more advanced disease of patients treated in third line. Although higher baseline levels lead to higher increases of CK18-Asp396, the increases do not vary as expressed as percent of baseline.

**Figure 2:**
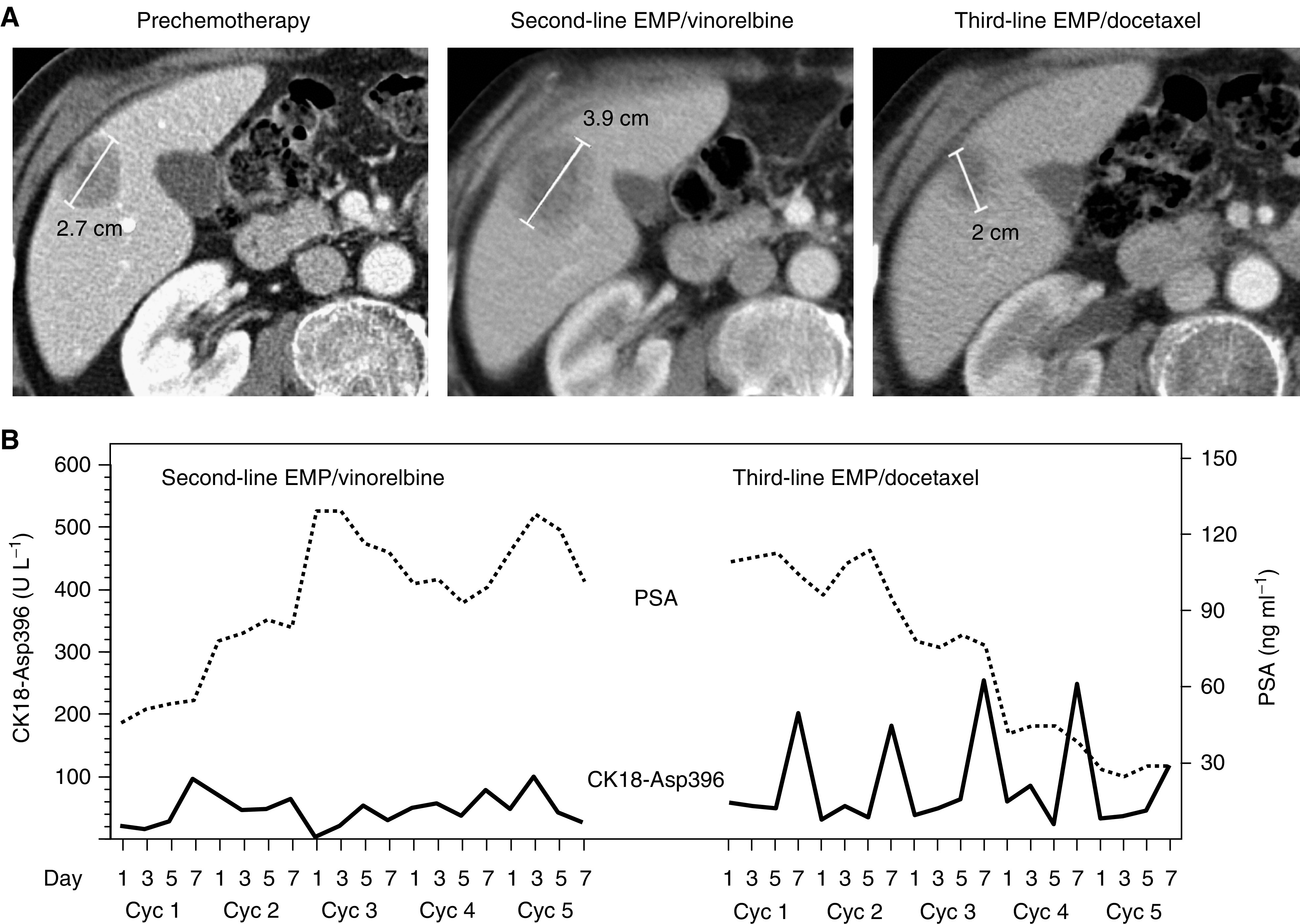
Levels of serum CK18-Asp396 in a patient with HRPC during second- and third-line chemotherapy. The patient (75 years old) had only detectable liver metastases (a reference lesion is shown in (**A**) by a multiphasic spiral CT scan with intravenous contrast media of the liver). In spite of five cycles of EMP/vinorelbine treatment in second line, liver metastases increased in number and size (before therapy shown in left panel of (**A**), after therapy in middle panel). Accordingly, serum PSA levels increased (dotted line in **B**). After switching to five cycles of EMP/docetaxel, a significant reduction in tumour volume (reference lesion shown in the right panel of (**A**)) and serum PSA levels was observed (**B**). Strong increases in CK18-Asp396 (solid line in (**B**)) were observed 48 h after docetaxel treatment (day 7) of these cycles.

Figure 2A shows a liver metastasis that increased in size from 2.7 to 3.9 cm during treatment with EMP and vinorelbine. Serum PSA levels increased in parallel (Figure 2B) and CK18-Asp396 increases during chemotherapy were low (Figure 2B). After changing therapy to EMP and docetaxel, the lesion decreased from 3.9 to 2 cm and PSA levels decreased dramatically. Stronger increases in CK18-Asp396 levels were observed during EMP and docetaxel therapy (Figure 2B).

### Patterns of CK18-Asp396 increases during therapy

Docetaxel was used as second-line, third-line and fourth-line therapy. Significant increases of serum CK18-Asp396 were observed irrespectively of whether docetaxel was used early or late during clinical progression (*P*<0.004, *P*<0.0002 and *P*<0.02 at second-, third- and fourth-line therapy, respectively; Wilcoxon matched-pairs signed-ranks test). Vinorelbine induced significant increase in CK18-Asp396 during second-line therapy (*P*<0.005), whereas the increases during third line were of borderline significance (*P*=0.048). Median increases are shown in Table 5.

We finally examined treatment-induced levels of CK18-Asp396 at different treatment cycles. Significant increases in CK18-Asp396 were observed during cycles 1–8 (Table 6). In contrast, significant increases in CK18-Asp396 were observed only during early vinorelbine treatment cycles (cycles 1–3), but not at later cycles (Table 6).

## DISCUSSION

Docetaxel and vinorelbine have previously been reported to induce apoptosis of human prostate cancer cells *in vitro* (Zelivianski *et al*, 2003). In contrast, apoptosis could not be demonstrated in a docetaxel-treated human prostate cancer xenograft (Oudard *et al*, 2003). We here report that both docetaxel and vinorelbine induce significant increases in serum CK18-Asp396 in HRPC patients. For docetaxel, the amplitudes of increases were associated with the baseline serum levels of PSA and CK18 prior to therapy. This association is not expected if increases are due to toxicity to normal epithelial cells, but are expected if the fragments are generated by apoptosis of tumour cells. The fraction of CK18 cleaved by caspases was approximately one-third of total CK18 both at baseline and after 48 h of therapy, suggesting that both basal and induced serum CK18 reflect apoptosis.

Similar to docetaxel, vinorelbine induced increases of CK18-Asp396 and CK18. In contrast to docetaxel, vinorelbine-induced increases in CK18 molecules were not significantly associated with baseline levels of PSA or CK18. Vinorelbine may therefore be speculated to induce toxicity to nontumour tissue. Alternatively, the increases in CK18 molecules do in fact represent tumour apoptosis, and the lack of association with basal level of PSA/CK18 is due to the relatively low increases in CK18-Asp396 induced by vinorelbine. Vinorelbine induced CK18-Asp396 increases during cycles 1–3, but not later at later cycles, a pattern that is difficult to explain by toxicity to normal cells. During cycles 1–3, we observed an association between CK18-Asp396 and baseline CK18 increases (lowest and highest CK18 quartile; *P*=0.015), but not to baseline PSA (corresponding *P*-value: 0.11). In distinction from docetaxel and vinorelbine, EMP did not induce increases in CK18-Asp396, but did induce increases in total CK18. Estramustine phosphate-induced CK18 increases were not associated with basal levels of PSA or CK18 and EMP did not show a clear pattern as to at which cycles the CK18 increases occurred (not shown). It is therefore possible that EMP-induced CK18 increases represent toxicity to normal tissue. Estramustine phosphate is almost exclusively used in combination with other drugs, and it is uncertain whether EMP has significant antitumour activity in these combinations (Kitamura *et al*, 2002). Estramustine phosphate has been tested in combination with docetaxel in a prostate carcinoma xenograft model, and was not found to increase the efficiency of docetaxel (Fizazi *et al*, 2004). Docetaxel and estramustine combinations induce significant cardiovascular and gastrointestinal toxicity, and the use of estramustine has been questioned (Armstrong and Carducci, 2005).

Prostate-specific antigen response rates of above 60% have been reported for EMP and docetaxel (Petrylak *et al*, 1999; Savarese *et al*, 2001), compared to 24% for EMP and vinorelbine (Smith *et al*, 2000). Recent studies have shown that docetaxel-based therapies lead to improved survival compared to mitoxantrone-based therapy (Petrylak *et al*, 2004; Tannock *et al*, 2004). We report significant increases in CK18-Asp396 during second-, third- and fouth-line docetaxel treatment, suggesting that the agent is effective in patients with advanced disease. In fact, higher CK18-Asp396 increases were observed during third-line docetaxel treatment compared to second-line vinorelbine treatment in the same group of 32 patients. The high efficiency of docetaxel appeared to be related to apoptosis induction during a number of consequtive treatment cycles (at least eight), compared to only three cycles for vinorelbine. It will be of interest to examine whether less-toxic docetaxel treatment modalities (lower doses given weekly) will induce similar levels of CK18-Asp396 as traditional schedules (Engels and Verweij, 2005).

The median increase in CK18-Asp396 during docetaxel therapy was 23 U l^−1^ (∼20% increase). The spread was considerable, with a 10th percentile of −22 U l^−1^ and a 90th percentile of 151 U l^−1^. The large individual variations in CK18-Asp396 increases are likely to be due to differences in apoptosis induction in different tumours. Whether such differences can be used to predict individual patient outcome is uncertain at this point. Chemotherapy has traditionally been used palliatively in HRPC, and although docetaxel has recently been demonstrated to confer a survival benefit in this disease, the increased survival time is only approximately 3 months (Petrylak *et al*, 2004; Tannock *et al*, 2004). It is unlikely that differences in apoptosis induction will be reflected in major differences in patient survival in this group of patients. In general, it is uncertain whether measurements of tumour cell death *in vivo* (by imaging, serum biomarkers or by other methods) will be strictly correlated to patient survival. Therapy may induce cell death of subpopulations of sensitive cells, whereas other cell populations continue to proliferate, leading to clinical failure. Nevertheless, the possibility to assess tumour apoptosis in serum of patients with advanced disease is very likely to be of considerable value for clinical decisions. Furthermore, these measurements are expected to be useful for demonstration of drug effects in early phase clinical trials.

Nucleosomes and cytochrome *c* represent other blood biomarkers for tissue damage (Holdenrieder and Stieber, 2004). These biomarkers are not released specifically from epithelial cells, and their levels in circulation are likely to reflect cell death of all chemotherapy-sensitive tissues. A recent study showed lower levels of circulating nucleosomal DNA in responding non-small lung cancer patients during chemotherapy (assessed as area under the curve between days 1 and 8) (Holdenrieder *et al*, 2004). We have not evaluated our data as an integrated measure of total release, but analysed increases during the course of treatment. An intriguing possibility is that combinations of blood biomarkers, which measure cell death in different cell populations (ideally tumour cells *vs* normal cells), can be used to achieve optimal dosing of anticancer drugs.

The present data suggest that serum CK18 measurements may be useful for assessing treatment effects. This method is inexpensive and sera can be frozen and stored before analysis (Cummings *et al*, 2005), making the method suitable for multicentre clinical trials. Measurements of caspase-cleaved CK18 in serum may be quite useful for comparisons of the efficiencies between different treatment modalities, which is a key issue during development of novel anticancer drugs.

## Figures and Tables

**Table 1:** Clinical parameters relevant to this study

	**All patients**	**EMP+vinorelbine**	**EMP+docetaxel**			
*Patient characteristics*						
Age of patients (years)	69 (48–88)	70 (48–87)	69 (49–88)			
Time from diagnosis to treatment (months)	66 (7–330)	65 (7–324)	68 (10–330)			
Lymph metastasis^a^	29 (36%)	21 (29%)	8 (88%)			
Bone metastasis^a^	44 (54%)	42 (58%)	2 (22%)			
Lung metastasis^a^	6 (7.3%)	1 (1.4%)	5 (55%)			
Liver metastasis^a^	5 (6.1%)	5 (6.8%)	0			
						

	**Second line**		**Third line**		**Fourth line**	
**Treatment**	**Patients**	**Cycles**	**Patients**	**Cycles**	**Patients**	**Cycles**
*Number of patients and therapy cycles analysed* b						
EMP+docetaxel	9	74	32	193	4	21
EMP+vinorelbine	62	404	11	54	1	5
						

	**Cycle 1**	**Cycle 2**	**Cycle 3**	**Cycle 4**	**Cycle 5**	
*PSA levels during consecutive treatment of 32 patients with vinorelbine and docetaxel (median (25th–75th percentile); ng ml^−1^)*						
Second line EMP+vinorelbine	68 (20–116) (100%)	47 (26–145) (69%)	49 (12–103) (72%)	29 (9–73) (43%)	33 (11–80) (49%)	
Third line EMP+docetaxel	104(27–162) (100%)	87 (18–132) (84%)	38 (11–107) (37%)	53 (24–123) (51%)	53 (23–173) (51%)	

a Presence of metastases at the initiation of chemotherapy.

b Total patients: 82 (several patients received more than one type of treatment).

EMP=estramustine phosphate; PSA=prostate-specific antigen.

**Table 2:** CK18 serum levels during chemotherapy


	**CK18-Asp396**		**CK18**	
**Day**	**(U l^−1^)**	**% Δ^a^**	**(U l^−1^)**	**% Δ**
*EMP (n=751 cycles)*				
1	102 (59–163)^b^	0	280 (169–457)	0
3	101 (63–158)	−1.0	303 (184–455)	8.2
				
*Docetaxel (n=289 cycles)*				
5	123 (86–171)	0	337 (227–514)	0
7	146 (101–229)	18.7	409 (263–595)	21.4
	(*P*=0.015)		(*P*=0.014)	
				
*Vinorelbine (n=462 cycles)*				
5	83 (53–135)	0	255 (165–419)	0
7	89 (58–159)	7.2	272 (166–451)	6.7

a Percent change: from day 1 to day 3 and from day 5 to day 7.

b Median (25th–75th percentile).

CK18=Cytokeratin 18; EMP=estramustine phosphate.

**Table 3:** Association between treatment-induced increases of CK18-Asp396 and CK18 and baseline levels of PSA, CK18 or CK18-Asp396

**Baseline PSA**	**CK18-Asp396 increase (U l^−1^) docetaxel**	**CK18 increase (U l^−1^) docetaxel**	**CK18-Asp396 increase (U l^−1^) vinorelbine**	**CK18 increase (U l^−1^) vinorelbine**	**CK18 increase (U l−1) EMP**
First quartile	13 (−4–50)	53 (−1–183)	7 (−4–36)	0 (−54–68)	23 (−21–87)
Second quartile	24 (2–66)	62 (4–148)	9 (−11–25)	7 (−46–85)	−1 (−83–53)
Third quartile	22 (−4–55)	50 (−16–187)	10 (−11–43)	13 (−50–90)	22 (−54–79)
Fourth quartile	82 (−23–126) *P*=0.02^a^	47 (−48–237)	7 (−3–35)	22 (−60–321) *P*=0.10	−7 (−144–124)
					
*Baseline CK18*					
First quartile	22 (0–53)	22 (−23–152)	7 (−10–26)	0 (−48–63)	30 (−14–86)
Second quartile	18 (0–53)	61 (−27–165)	6 (−8–20)	20 (−14–90)	11 (−33–73)
Third quartile	13 (−13–47)	21 (−19–121)	14 (−2–45)	7 (−46–86)	0 (−84–62)
Fourth quartile	92 (15–169) *P*=0.0002	136 (10–423) *P*=0.006	7 (−6–49)	24 (−96–130) *P*=0.31	−59 (−251–124)
					
*Baseline CK18-Asp396*					
First quartile	13 (−1–43)	16 (−30–108)	7 (−6–19)	4 (−52–64)	29 (−28–83)
Second quartile	11 (−4–46)	56 (−34–123)	7 (−8–31)	3 (−37–95)	2 (−54–67)
Third quartile	42 (0–91)	97 (1– 216)	6 (−7–31)	12 (−57–104)	−2 (−110–75)
Fourth quartile	54 (1–151) *P*=0.004	87 (−3–232) *P*=0.06	16 (−7–81) *P*=0.08	38 (−69–136) *P*=0.15	14 (−116–117)

a Wilcoxon Two-Sample test; comparing the median level of the lowest and highest quartile.

CK18=Cytokeratin 18; PSA=prostate-specific antigen.

**Table 4:** Alterations of CK18-Asp396 in patients first treated with EMP/vinorelbine and then with EMP/docetaxel

**Increase in CK18-Asp396**				**Increase in CK18**			
**Second vinorelbine**		**Third docetaxel**		**Second vinorelbine**		**Third docetaxel**	
**(days 1–3)**	**(days 5–7)**	**(days 1–3)**	**(days 5–7)**	**(days 1–3)**	**(days 5–7)**	**(days 1–3)**	**(days 5–7)**
2 (−27–14)^a^	5 (−11–29)	6 (−27–44)	22 (−1–54)	9 (−58–67)	0 (−47–68)	9 (−67–79)	50 (−4–76)
2.7%^b^	6.7%	5.1%	18.6%	4.1%	0%	2.8%	15.7%

a Alterations expressed in U l^−1^.

b Alterations expressed as percent of base line at day 1 or 5.

CK18=Cytokeratin 18; EMP=estramustine phosphate.

**Table 5:** Alterations of CK18-Asp396 during second-, third- and fourth-line docetaxel therapy

	**CK18-Asp396 increase (days 5–7)**		**CK18 increase (days 5–7)**		
	**(U l^−1^)**	**Percent increase**	**(U l^−1^)**	**Percent increase**	**(***n***^a^)**
Second docetaxel	49 (0–115)^b^	31.7	93 (−21–236)	18.3	54/54
Third docetaxel	22 (0–54)	16.1	47 (−4–163)	15.0	154/155
Fourth docetaxel	34 (13–98)	29.3	72 (2–202)	30.8	14/15

a (CK18-Asp396/CK18).

b Median (25th–75th percentile) alterations from day 5 to day 7.

CK18=Cytokeratin 18.

**Table 6:** Increases in CK18-Asp396 during different treatment cycles

**Docetaxel (days 5–7)**					**Vinorelbine (days 5–7)**			
**Cycle^a^**	**CK18-Asp396 increase (Ul^−1^)**	**Percent increase**	**(*n*)**	***P***-**value^a^**	**CK18-Asp396 increase (Ul^−1^)**	**Percent increase**	**(*n*)**	***P***-**value^a^**
1	27 (−5–91)	23.3	43	<0.005	13 (0–51)	11.6	66	0.008
2	29 (−7–86)	22.1	42	<0.013	8 (−10–28)	9.1	65	0.01
3	21 (−2–72)	18.4	35	<0.011	14 (0–40)	17.9	60	0.03
4	36 (4–70)	30.5	33	<0.0004	0 (−18–28)	0	57	0.70
5	36 (1–68)	27.1	31	<0.0004	2 (−10–19)	2.5	51	0.38
6	33 (0–110)	28.4	24	<0.007	0 (−31–53)	0	36	0.64
7	9 (0–48)	7.7	22	<0.022	6 (−22–32)	7.1	29	0.52
8	19 (−7–46)	15.8	19	<0.031	5 (−13–14)	6.8	23	0.54

Of 567 cycles assessed for increases during days 5–7, 181 samples were available from docetaxel cycles 1–8; 294 from vinorelbine cycles 1–8.

a Wilcoxon matched-pairs test.

CK18=Cytokeratin 18.
